# Oscillatory zoning of minerals as a fingerprint of impurity-mediated growth

**DOI:** 10.1038/s41598-024-63722-4

**Published:** 2024-06-20

**Authors:** Hiroki Torii, Hitoshi Miura

**Affiliations:** Graduate School of Science, Nagoya City University, Yamanohata 1, Mizuho-cho, Mizuho-ku, Nagoya, Aichi 4678501 Japan

**Keywords:** Geophysics, Mineralogy

## Abstract

We propose a kinetic mathematical model of the oscillatory compositional zoning profile recorded in minerals based on the crystal growth suppression induced by impurities. Notably, the presence of a small amount of impurities significantly inhibits crystal growth, and a growth inhibition mechanism called the pinning effect is widely accepted. Here we show that a model that considers the pinning effect and adsorption/desorption kinetics of impurities on the crystal surface can reproduce the oscillatory compositional zoning. As impurities are common in nature, this model suggests the existence of a universal mechanism that can occur in the growth processes of various crystals.

A zonal structure is observed on mineral sections wherein different compositions show nearly parallel distributions. Among these structures, those wherein compositional changes are periodically repeated are called oscillatory compositional zoning (OCZ). Plagioclase, a major rock-forming mineral, is a notable example exhibiting OCZ^1^. Plagioclase is a solid solution with anorthite (An; CaAl_2_Si_2_O_8_) and albite (Ab; NaAlSi_3_O_8_) as end members, and the mole fraction of An varies periodically. OCZs have also been identified in Ti in quartz^2^ and trace elements, such as P and Al, in olivine^3^. OCZs have also been observed in pyroxene for major and trace elements^4^. Elucidating the conditions and mechanisms of OCZ formation is important for deciphering the formation history of these minerals.

The cause of OCZ can be classified into two main categories: external (periodic fluctuations of external factors, such as temperature, composition, and pressure) and intrinsic (mechanisms inherent in the crystal growth process itself). Here, we focus on the latter, especially on the crystal growth in solution. In the intrinsic mechanism, the influence of a compositional boundary layer formed around the growing mineral is considered^5,6^. The elemental partitioning between the mineral and ambient solution causes compositional changes in the boundary layer and subsequently changes the driving force of crystallization at the mineral surface. The formation of OCZ requires a nonlinear relationship between the driving force and crystal growth rate. Three main models have been proposed to explain this nonlinear relationship. The first model is called surface reconstruction, wherein the crystal growth rate depends on the microstructure of the crystal surface, but the microstructure does not respond immediately to changes in the driving force^7–9^. The second model is mainly applied to plagioclase, wherein the rate of incorporation of An or Ab into plagioclase depends on the mole fraction of each component on the surface^10,11^. The third model considers non-equilibrium elemental partitioning at the crystal surface and is based on the assumption that the partition coefficients are reversed from those at equilibrium^12–14^.

In this study, we focus on the effect of impurities on crystal growth, which has not been considered in the aforementioned models, and theoretically show that the general crystal growth inhibition effect induced by impurities causes OCZ. Here, impurities are defined as components other than the major crystal constituents present in the solution.

## Crystal growth hysteresis induced by impurities

**Figure 1:**
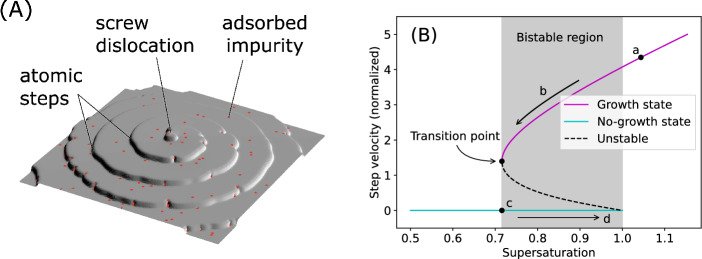
Mechanism of growth inhibition by impurities adsorbed on the crystal surface. (**A**) Interaction between adsorbed impurities and atomic steps. The crystal surface has a step, which is as high as one atom, and the continuous incorporation of atoms into this step moves the step forward and builds the surface layer by layer (layer growth). The step is generated by screw dislocations exposed to the surface. Adsorbed impurities inhibit the step advancement by pinning it in place (pinning effect). (**B**) Steady-state solution of the step velocity when considering impurities that are repeatedly adsorbed and desorbed on the surface. The abscissa and ordinate represent the degree of supersaturation and dimensionless step velocity, respectively. In an intermediate supersaturation region (bistability region), there exist two solutions: growth (purple) and no-growth (blue) states. The dashed curve indicates an unstable solution that does not appear in practice.

Crystal surface is stacked in layers by atomic steps moving forward, generally generated by screw dislocations exposed to the surface^15^. Impurities are adsorbed on the growing crystal surface and inhibit its growth by checking the advancement of these steps (Fig. 1A). This is called the step pinning effect and has been widely confirmed in the growth process of various crystals^16^. Conversely, the impurity adsorption rate is known to be finite, which causes complex nonlinear phenomena owing to competition with the crystal growth rate. One theory that explains this nonlinear phenomenon is the bistability of the growth rate^17–20^. Figure 1B shows the dependence of the growth rate on the degree of supersaturation (see Methods for details). At high supersaturation, the crystal surface is kept renewed with new successive steps before it is covered with impurities; thus, few adsorbed impurities are present and the crystal growth is hardly hindered (point a). As the supersaturation decreases, the frequency of surface renewal decreases, adsorbed impurities gradually increase, and the growth rate decreases (path b). When the supersaturation falls below a certain critical value, the step advancement stops completely owing to positive feedback as the surface that is no longer renewed is further contaminated by impurities (point c). This surface is saturated by adsorbed impurities (a state where adsorption and desorption of impurities are balanced, namely, adsorption/desorption equilibrium); thus, the crystal growth does not resume immediately even if supersaturation begins to increase (path d). However, once the growth resumes, the impurity-free surface is restored (point a). The property wherein multiple states can be realized in the intermediate supersaturation region is called bistability, and it explains the hysteresis phenomenon wherein different growth histories are followed by an increase or decrease in supersaturation.

## Oscillataory zoning

**Figure 2:**
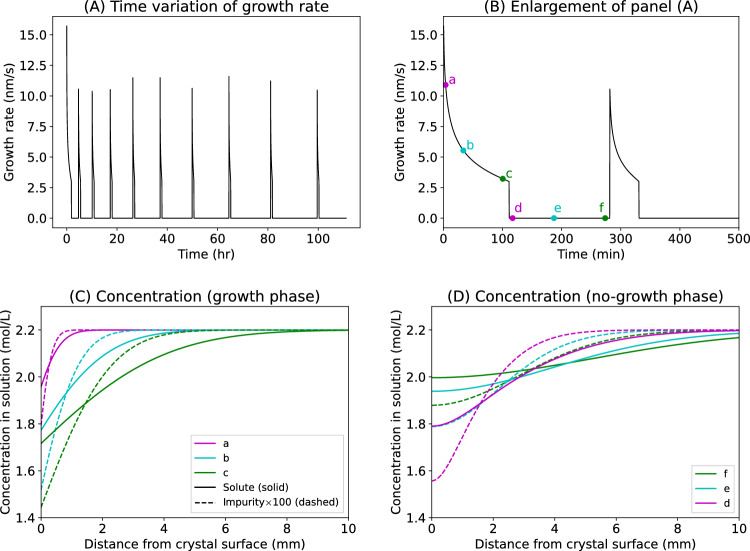
Calculated normal growth rate and concentration distributions in solution. (**A**) Time variation of the normal growth rate. (**B**) Enlargement of abscissa of panel A. (**C**) Time variation of concentration distributions in the solution during crystal growth. The solid and dashed lines indicate the solute and impurity concentration distributions, respectively. The impurity concentration is multiplied by a factor of 100 for comparison with the solute concentrations. Labels a–c correspond to those in panel B. (**D**) Time variation of the concentration distributions when the crystal stops growing. Labels d–f correspond to those in panel B. In this calculation, impurity diffusion is assumed to be slower than the solute diffusion.

We conducted simple numerical calculations considering elemental diffusion in the solution to show that the impurity-induced crystal growth hysteresis causes OCZ. Assuming that the crystal surface is an infinitely wide plane, the time-related variation of the concentration distribution of solute and impurity molecules in the solution is obtained by solving the diffusion equation, and the relationship between the interfacial supersaturation and crystal growth rate is elucidated based on the growth hysteresis theory (see Methods for details). The time-related variation of the normal growth rate (the crystal growth rate perpendicular to the crystal surface) obtained from the numerical calculation is shown in Fig. 2A,B. Notably, the growth rate periodically increases and decreases. Figure 2C,D show the time-related variation of the concentration distribution in the solution during crystal growth and growth stoppage, respectively. Notably, a compositional boundary layer is developed near the crystal surface; the concentrations of both the solute and impurity decrease during growth, and are then recovered by diffusive transport from the bulk when the growth is terminated.

**Figure 3:**
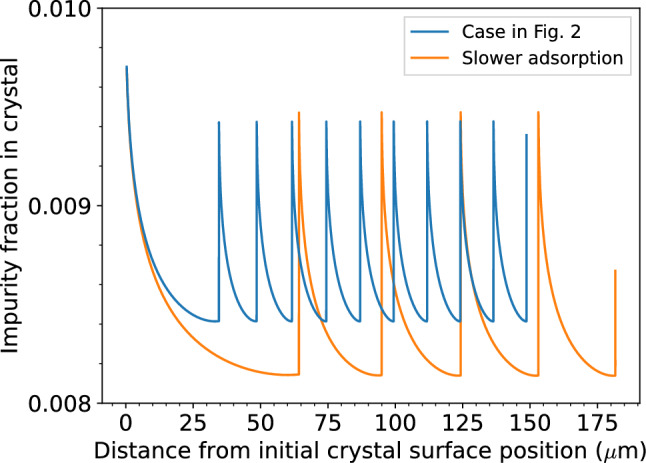
Impurity distribution in the grown crystal. The abscissa represents the distance from the initial crystal surface position, and the ordinate represents the impurity fraction relative to solute molecules. The blue line shows the case in Fig. 2. The orange line shows the case when the impurity adsorption is assumed to be slower.

Based on above results, we calculated the compositional zoning profile in the grown crystal. The impurity fraction with respect to the solute incorporated into the as-grown surface is proportional to the impurity fraction in the solution. In this study, the impurity fraction in the solution near the crystal surface was multiplied by the constant *K* to obtain that in the grown crystal at that time (see Methods for details). *K* is a quantity commonly referred to as the partition coefficient. The resultant zoning profile is shown in Fig. 3 with the blue line. Immediately after the start of crystal growth, the impurity fraction decreases with crystal growth. This is because the impurity diffusion in the solution is assumed to be slower than the solute diffusion in this calculation, so the impurities in the compositional boundary layer are more depleted with respect to the solute molecules. When the crystal growth is then stopped, the impurity fraction increases as the impurities depleted in the boundary layer are supplied by diffusive transport. Thus, the impurity fraction in the crystal at the time of re-growth is higher than that immediately before the termination of growth. Therefore, the impurity fraction in the crystal changes periodically; namely, OCZ is developed.

The characteristics of OCZ, such as interval (wavelength) and amplitude of compositional fluctuations, depend on the impurity adsorption kinetics. For comparison, we demonstrate the calculation for the case where the density of adsorbed impurities at adsorption/desorption equilibrium is the same, but when a longer time is required to reach equilibrium (orange line in Fig. 3). OCZ was reproduced but the wavelength was longer than that of the case when the impurity adsorption is faster. In the case of slow adsorption, the crystal surface is less likely to be contaminated by impurities, so its growth can be maintained until the supersaturation becomes lower. Therefore, the wavelength is increased as the crystal can grow for a longer time before being terminated. This indicates that the wavelength of OCZ not only depends upon the diffusion coefficient in the solution but also on the behavior of elements on the crystal surface, suggesting that more careful consideration is required in interpreting the formation condition of OCZs.

The present study shows that OCZs can occur under typical solution growth conditions (see Methods for details). We present qualitative predictions for cases where OCZs do not occur. The first case is when crystal growth is not controlled by diffusion. In our model, growth oscillation is triggered by local changes in solute concentration in the boundary layer near the crystal surface. Therefore, the proposed mechanism is ineffective in systems where (1) uptake at the crystal surface determines the growth rate; (2) the free space between adjacent crystals is sufficiently small compared to the diffusion length; or (3) the boundary layer is disturbed by convection. The second case is when the bistability region is sufficiently narrow. Our model assumes the existence of the bistability region (Fig. 1B), and predicts its width (see Methods for details). The bistability region is expected to be very narrow in systems where (1) crystals are made of poorly soluble materials; (2) crystal growth is controlled by the surface incorporation process; (3) the desorption/adsorption of impurities is extremely rapid; (4) the amount of impurities adsorbed on the crystal surface is limited; or (5) the adsorbed impurities are incompletely removed by a single step passage. In such systems, the proposed mechanism will be ineffective as the bistability region only appears under limited supersaturation conditions. The desorption/adsorption behavior of impurities on crystal surfaces is often poorly understood, and it is difficult to compare our model directly with experimental data, however the qualitative predictions discussed herein is usable to validate our model.

## Conclusions and outlook

As many impurities are present in nature, the impurity-induced growth hysteresis can occur in general. OCZs found in minerals, such as quartz, olivine, and pyroxene^2–4^, may be the result of incompatible elements that are not easily incorporated into minerals playing the role of impurities. The periodic fluctuation of An content in plagioclase^1^ may be ascribed to the growth-inhibiting effect of impurities, rather than to the previously proposed growth kinetics unique to binary systems^10,11^. In addition to those of these minerals, the oscillatory behaviours and the periodic structures have been reported in various crystal growth systems that may be attributed to the action of impurities. The growth hysteresis and spontaneous oscillatory growth observed in ice crystal growth experiments are also thought to be related to the effect of proteins added as impurities in the system^21–24^. Recently, calcium-binding proteins have been found to be periodically distributed in human kidney stones, which are mainly composed of calcium oxalate, and the effect of these proteins on the stone growth has garnered considerable attention^25–27^. The impurity effects on crystal growth discussed in this study may be a common cause of the oscillatory phenomena and periodic structures observed in various crystals.

## Methods

### Bistability theory of crystal growth hysteresis

The bistability of the step velocity in the presence of impurities adsorbed on the crystal surface has been studied theoretically^17,18^ and numerically^19,20^. Figure 1A shows the schematic of a crystal surface undergoing spiral growth. The spiral step, centered on a screw dislocation, moves forward, building the crystal surface layer by layer. The step interval is given by $\lambda = 19 \rho _\mathrm{c}$, where $\rho _\mathrm{c} = \frac{s_\mathrm{m} \kappa }{k_\mathrm{B} T \sigma }$ is the radius of a two-dimensional critical nucleus^28^. $s_\mathrm{m}$ is the area of the crystal surface occupied by a growth unit, $\kappa$ is the step-edge free energy, $k_\mathrm{B}$ is the Boltzmann constant, *T* is the absolute temperature, and $\sigma$ is the supersaturation. If the step velocity is $V_\mathrm{i}$, the crystal surface is updated on average with the period $\tau _\mathrm{update} = \lambda /V_\mathrm{i}$. However, impurities in the solution are adsorbed onto the crystal surface at a certain rate. A faster adsorption process causes the crystal surface to be constantly contaminated (adsorption/desorption equilibrium). Conversely, if the adsorption is slow, the crystal surface is successively renewed before impurities are adsorbed again, thus maintaining a clean surface. This suggests that the density of adsorbed impurities $N_\mathrm{i}$ (number per unit area) depends on $V_\mathrm{i}$ and $\lambda$. At the same time, $V_\mathrm{i}$ depends on $N_\mathrm{i}$ as the steps are prevented from advancing by the adsorbed impurities. The interdependence between $V_\mathrm{i}$ and $N_\mathrm{i}$ clarifies the emergence of crystal growth hysteresis. Herein we outline the theory of growth hysteresis based on the Miura formulation^20^.

The step moves forward by slipping through the gaps between the adsorbed impurity molecules. During this, the steps are bent and velocity is reduced. This mechanism of crystal growth inhibition is widely accepted as the pinning mechanism^16^. The average step velocity $V_\mathrm{i}$ is as follows^29^.

1 $$\begin{aligned} V_\mathrm{i} = V_{\infty } \sqrt{1 - 2 \rho _\mathrm{c} N_\mathrm{i}^{1/2}} \end{aligned}$$

where $V_{\infty }$ is the velocity of a straight step when the effect of impurities is negligible and proportional to supersaturation, expressed as $V_{\infty } = \beta _\mathrm{st} v_\mathrm{m} c_\mathrm{e} \sigma$. $\beta _\mathrm{st}$ is the step kinetic coefficient, $v_\mathrm{m}$ is the molar volume of the solute molecules, and $c_\mathrm{e}$ is the solubility^30^. We use a coefficient $V_0 = \beta _\mathrm{st} v_\mathrm{m} c_\mathrm{e}$ to denote $V_{\infty } = V_0 \sigma$. The interior of the square root becomes negative when $N_\mathrm{i} > 1/(2 \rho _\mathrm{c})^2$, whereby step advancement is completely inhibited ($V_\mathrm{i}=0$).

When the step passes, the crystal surface is updated and $N_\mathrm{i}$ is reset to zero. Subsequently, the adsorbed impurities increase with time. The adsorbed impurity density when another step passes is expressed as a solution of the time-dependent Langmuir adsorption equation as follows.

2 $$\begin{aligned} N_\mathrm{i} = N_\mathrm{e} (1-e^{-\tau _\mathrm{update}/\tau _\mathrm{ad}}) = N_\mathrm{e} (1-e^{-\lambda / V_\mathrm{i} \tau _\mathrm{ad}}) \end{aligned}$$

where $N_\mathrm{e}$ is the value of $N_\mathrm{i}$ at the adsorption/desorption equilibrium and $\tau _\mathrm{ad}$ is the time needed to reach the adsorption/desorption equilibrium (adsorption time). If the update frequency is high ($\tau _\mathrm{update}/\tau _\mathrm{ad} \ll 1$), we obtain $N_\mathrm{i} / N_\mathrm{e} \rightarrow 0$, indicating that the crystal surface is updated before reaching the adsorption/desorption equilibrium. If the update frequency is low or crystal growth has stopped ($\tau _\mathrm{update}/\tau _\mathrm{ad} \gg 1$), we obtain $N_\mathrm{i} / N_\mathrm{e} \rightarrow 1$, indicating that the crystal surface is contaminated with impurities until the adsorption/desorption equilibrium is reached.

Equations (1) and (2) formulate the interdependence between $V_\mathrm{i}$ and $N_\mathrm{i}$. As the crystal steadily grows, these two equations are satisfied, this is called the steady-state solution. To identify the factors that determine the steady-state solution, we make these equations dimensionless. The step velocity is normalized as $\hat{V}_\mathrm{i} = V_\mathrm{i} / (\lambda _0/\tau _\mathrm{ad})$ and $\hat{V}_0 = V_0 / (\lambda _0/\tau _\mathrm{ad})$, where $\lambda _0$ is the step interval when $\sigma = 1$ and is defined by $\lambda = \lambda _0 / \sigma$. The density of the adsorbed impurities is normalized as $\theta = N_\mathrm{i} / N_\mathrm{e}$. Using these normalized quantities, Eqs. (1) and (2) are rewritten as follows.

3 $$\begin{aligned}{} & {} \hat{V}_\mathrm{i} = \hat{V}_0 \sigma \left( 1 - \frac{\hat{\kappa } \bar{\theta }^{1/2}}{\sigma } \right) ^{1/2} \end{aligned}$$

4 $$\begin{aligned}{} & {} \bar{\theta } = 1 - \hat{V}_\mathrm{i} \sigma (1 - e^{-1/\hat{V}_\mathrm{i} \sigma }) \end{aligned}$$

where $\hat{\kappa } = \frac{2 s_\mathrm{m} N_\mathrm{e}^{1/2} \kappa }{k_\mathrm{B}T}$ is the normalized step-edge free energy. $\bar{\theta }$ is the value obtained by time averaging Eq. (2) from immediately after the step pass to the next step pass. $\hat{V}_\mathrm{i} = 0$ when $\bar{\theta } = 1$ and $\sigma < \hat{\kappa }$ (Eq. 3). Thus, $\hat{\kappa }$ corresponds to the minimum supersaturation required for a crystal surface that has stopped growing to start growing again. The relationship between $\hat{V}_\mathrm{i}$ and $\bar{\theta }$ for the three cases with different supersaturations is shown in Supplementary Figure 1. When $\sigma = 0.6$, Eqs. (3) and (4) have an intersection a with $\hat{V}_\mathrm{i} = 0$, indicating that step advancement is completely inhibited (no-growth solution). When $\sigma = 1.1$, there is an intersection b with $\hat{V}_\mathrm{i} > 0$, indicating that the step continues to advance (growth solution). Multiple intersections exist, a, c, and d, when $\sigma = 0.9$. Thus, a solution is chosen according to previous growth history. By numerically solving Eqs. (3) and (4) for the two given dimensionless quantities $\hat{V}_0$ and $\hat{\kappa }$, we can obtain $\hat{V}_\mathrm{i}$ in the steady growth solution.

Figure 1B shows the calculation results for the case with $\hat{V}_0 = 5$ and $\hat{\kappa } = 1$. See the next section for the detailed calculation method. Only the growth solution exists when $\sigma > \hat{\kappa }$, and only the no-growth solution exists when $\sigma < 0.716$. When $0.716< \sigma < \hat{\kappa }$, both solutions exist (bistability region), thus, the preferred solution depends on previous growth history.

### Calculation of steady solution

The point where the growth solution disappears is called the transition point, and the supersaturation and step velocity at this point are $\sigma _*$ and $\hat{V}_*$, respectively. If $p = \sigma _*/\hat{\kappa }$, $q = \hat{V}_* \sigma _*$, and $u = \hat{V}_*/\hat{V}_0 \sigma _*$, then the following holds^20^.

5 $$\begin{aligned}{} & {} 4 p^2 u^2 (1-u^2) + (1+q)e^{-1/q} - q = 0 \end{aligned}$$

6 $$\begin{aligned}{} & {} u^2 = 1 - \frac{\sqrt{1-q(1-e^{-1/q})}}{p} \end{aligned}$$

7 $$\begin{aligned}{} & {} p^2 = \frac{q/u}{\hat{V}_0 \hat{\kappa }^2} \end{aligned}$$

Substituting Eq. (7) into Eqs. (5) and (6) to eliminate *p*, we obtain the following.

8 $$\begin{aligned} f(u,q)\equiv & {} \frac{4}{\hat{V}_0 \hat{\kappa }^2} q u (1-u^2) + (1+q) e^{-1/q} - q = 0 \end{aligned}$$

9 $$\begin{aligned} g(u,q)\equiv & {} \hat{V}_0 \hat{\kappa }^2 u \left[ 1 - q(1-e^{-1/q}) \right] - q(1-u^2)^2 = 0 \end{aligned}$$

If *u* and *q* are calculated by numerically solving Eqs. (8) and (9) using the Newton method, we obtain the variables at the transition point as $\sigma _* = \sqrt{\frac{q/u}{\hat{V}_0}}$ and $\hat{V}_* = q/\sigma _*$. When $\hat{V}_0 = 5$ and $\hat{\kappa } = 1$, we obtain $\sigma _* = 0.71566$ and $\hat{V}_* = 1.39596$.

The step velocity $\hat{V}_\mathrm{i}$ has multiple solutions when $\sigma _*< \sigma < \hat{\kappa }$ (Fig. 1B). The preferred steady-state solution depends on previous growth history. If the crystal is growing, a growth solution is realized when $\sigma \ge \sigma _*$, and a no-growth solution when $\sigma < \sigma _*$. However, when crystal growth is halted, a no-growth solution is realized when $\sigma \le \hat{\kappa }$ and a growth solution when $\sigma > \hat{\kappa }$. The value of $\hat{V}_\mathrm{i}$ at the growth solution is obtained using the nonlinear equation, which is obtained by eliminating $\bar{\theta }$ from Eqs. (3) and (4).

10 $$\begin{aligned} \left( \frac{\hat{V}_\mathrm{i}}{\hat{V}_0 \sigma } \right) ^2 = 1 - \frac{\hat{\kappa }}{\sigma } \left[ 1 - \hat{V}_\mathrm{i} \sigma (1-e^{-1/\hat{V}_\mathrm{i} \sigma }) \right] ^{1/2} \end{aligned}$$

The solution to this equation, $\hat{V}_\mathrm{i}$, is obtained by numerically searching between $\hat{V}_*$ and $\hat{V}_{\infty }$ using the bisection scheme.

### Diffusion of solute and impurity molecules in solution

We assumed that the crystal–solution interface is an infinitely wide plane and is present on the *x*-axis from the interface perpendicularly toward the solution. The molar concentration distribution of the solute and impurity molecules in the solution at a certain time *t* is denoted by $c_{\alpha } (x,t)$, where $\alpha$ denotes the type of molecule, and the solute and impurity are denoted by $\alpha = \textrm{s}$ and $\alpha = \textrm{i}$, respectively. The solute and impurity are depleted near the interface by being incorporated into the growing crystal and then supplied by diffusion from the far side. The convective transport is ignored. The time variation of $c_{\alpha }$ is expressed by the following unsteady one-dimensional diffusion equation:

11 $$\begin{aligned} \frac{\partial c_{\alpha }}{\partial t} = D_{\alpha } \frac{\partial ^2 c_{\alpha }}{\partial x^2} , \end{aligned}$$

where $D_{\alpha }$ is the diffusion coefficient. We assumed that the crystal growth is sufficiently slow and that the movement of the interface does not affect $c_{\alpha }$. In addition, when solving Eq. (11), the position of the interface is always assumed to be $x=0$.

The boundary condition at the interface is determined by the conservation law for each molecule. The number of moles of molecules flowing into the interface from the solution per unit area and unit time (interfacial flux) is given by Fick’s law of diffusion: $j_{\alpha } = D_{\alpha } \frac{\partial c_{\alpha }}{\partial x}$. If the impurity fraction with respect to the solute is sufficiently small, we can assume that the crystal thickens by the amount of solute incorporated into the interface. The relationship between the normal growth rate *R* (the crystal growth rate perpendicular to the interface) and interfacial flux of solute $j_\mathrm{s}$ is expressed as $R = v_\mathrm{m} j_\mathrm{s}$. Thus, we can obtain the following boundary condition for solute molecules:

12 $$\begin{aligned} D_\mathrm{s} \frac{\partial c_\mathrm{s}}{\partial x} = \frac{R}{v_\mathrm{m}} . \quad (\textrm{at} ~ x=0) \end{aligned}$$

Conversely, the amount of impurity molecules incorporated into the crystal is determined using the partition coefficient *K*, which is the ratio of the impurity fraction in the crystal to that in the solution. The impurity fraction in the solution near the interface is expressed as $c_\mathrm{i}(0,t)/c_\mathrm{s}(0,t)$, and that in the grown crystal is expressed as $j_\mathrm{i}/j_\mathrm{s}$. Therefore, the distribution coefficient is expressed as $K = \frac{j_\mathrm{i}/j_\mathrm{s}}{c_\mathrm{i}(0,t)/c_\mathrm{s}(0,t)}$. Thus, we can obtain the following boundary condition for impurity molecules:

13 $$\begin{aligned} D_\mathrm{i} \frac{\partial c_\mathrm{i}}{\partial x} = K \frac{c_\mathrm{i}(0,t)}{c_\mathrm{s}(0,t)} \frac{R}{v_\mathrm{m}} . \quad (\textrm{at} ~ x=0) \end{aligned}$$

In this study, we considered a closed system. The size of the computational domain is set to *L* and the no-flux boundary condition $\frac{\partial c_{\alpha }}{\partial x} = 0$ is imposed at $x = L$. *L* was set to be large enough to ensure that the effect of the boundary layer does not reach it.

### Calculation procedure

The interfacial supersaturation was calculated as $\sigma = \frac{c_\mathrm{s}(0,t)-c_\mathrm{e}}{c_\mathrm{e}}$, where $c_\mathrm{e}$ is the solubility. As two solutions of $\hat{V}$ exist when $\sigma _*< \sigma < \hat{\kappa }$, we used a logical variable *w*, which represents the growth state of the crystal surface, to determine which solution is chosen. The initial value of *w* was set as “True;” changed to “False” when $\sigma$ fell below $\sigma _*$ and to “True” when $\sigma$ exceeded $\hat{\kappa }$. When *w* was “True,” we solve Eq. (10) to obtain $\hat{V}_\mathrm{i}$, and when it was “False,” we just set $\hat{V}=0$. The normal growth rate was determined using $R = (a/\lambda ) V_\mathrm{i} = (a/\tau _\mathrm{ad}) \sigma \hat{V}_\mathrm{i}$, where *a* is the step height. Note that the calculation result does not depend on the value of $\lambda _0$. *R* was substituted into the boundary conditions (Eqs. 12 and 13) and the diffusion equation (11) was solved numerically to obtain the time variation of the concentration distributions $c_\alpha (x,t)$. The computational domain from $x=0$ to *L* was divided by cells with equal intervals of $\Delta x$, and Eq. (11) was solved using the finite volume method. The time increment was set to $\Delta t$, and the first-order upwind difference was used for the time derivative. By repeating these procedures, we obtained the time variations of *R*(*t*) and $c_{\alpha }(x,t)$. The growth thickness is given by $h(t) = \int _0^t R(t') dt'$ and the impurity fraction of the grown crystal surface is given by $\chi (t) \equiv \frac{j_\mathrm{i}}{j_\mathrm{s}} = K \frac{c_\mathrm{i}(0,t)}{c_\mathrm{s}(0,t)}$, so the impurity distribution in the grown crystal is shown in the plot of *h*(*t*) versus $\chi (t)$.

The values of the parameters used in the calculations are summarized in Supplementary Table 1. Herein, typical solution growth situations were considered. The diffusion coefficients of the solute molecules were taken from typical values for metallic ions in aqueous solution^31^. Impurity molecules were assumed to be larger than solute molecules and their diffusion was assumed to be slower than that of solute molecules. For solubility, typical values for soluble substances in water were used^32^. The initial solute concentration was set sufficiently high to allow crystal growth. The initial concentration of impurity molecules was 1/100 of the solute molecules. The adsorption time $\tau _\mathrm{ad}$ of the impurity molecules on the growing crystal surface is not well-known. The adsorption time of macromolecules, such as proteins, on inorganic crystal surfaces varies from less than one minute to several tens of minutes, depending on the inorganic crystal and impurity molecules^16^. Analyzing ice crystal growth hysteresis with synthetic antifreeze proteins has revealed extremely short adsorption times of $< 0.1$ s^21^. With reference to the literature, the case of $\tau _\mathrm{ad} = 0.1 \ \textrm{s}$ is considered in this study. For the inorganic crystals, the step kinetic coefficient is estimated as $\beta _\mathrm{st} \sim 10^{-2}$–$10^{-1} \ \mathrm {cm/s}$^30^; thus, $V_0 = \beta _\mathrm{st} v_\mathrm{m} c_\mathrm{e} \sim 10^{-5} \ \mathrm {m/s}$. Assuming that the step interval is $\lambda _0 \sim 100 \ \textrm{nm}$, $\hat{V}_0 = V_0 / (\lambda _0 / \tau _\mathrm{ad}) \sim 10$. Thus, we considered the case where $\hat{V}_0 = 5$. Assuming that the halted growth of a crystal will resume when $\sigma > 1$, we set $\hat{\kappa } = 1$. *L* was sufficiently large to discontinue the influence of the boundary layer on the outer edge of the computational domain. Our findings for the slow impurity adsorption ($\tau _\mathrm{ad} = 0.2 \ \textrm{s}$ and $\hat{V}_0 = 10$) case are shown in Fig. 3, confirming its dependence on impurity adsorption kinetics.

### Width of the bistability region

The supersaturation $\sigma _*$ at the transition point is dependent on the value of $\hat{V}_0 \hat{\kappa }^2$ (Eqs. 8 and 9). For $\hat{V}_0 \hat{\kappa }^2 < 1$, $\sigma _* / \hat{\kappa }$ is almost 1^20^, indicating that the bistability region becomes very narrow, and therefore growth hysteresis is unlikely to occur. Here we have

14 $$\begin{aligned} \hat{V}_0 \hat{\kappa }^2 = \frac{\beta _\mathrm{st} v_\mathrm{m} c_\mathrm{e}}{\lambda _0 / \tau _\mathrm{ad}} \frac{4 s_\mathrm{m}^2 N_\mathrm{e} \kappa ^2}{(k_\mathrm{B}T)^2} . \end{aligned}$$

The cases where the value of $\hat{V}_0 \hat{\kappa }^2$ is small are as follows. First, $c_\mathrm{e}$ is small (insoluble), $\tau _\mathrm{ad}$ is small, or $\beta _\mathrm{st}$ is small. This corresponds to a situation where crystal growth is slower than impurity adsorption, and adsorption/desorption equilibrium is always reached at the crystal surface, thus, the interdependence between step velocity and adsorbed impurity density is untenable. Second, $N_\mathrm{e}$ is small and step advancement cannot be halted at intermediate supersaturation conditions because the adsorbed impurity density is low at the adsorption/desorption equilibrium. Hence, the bistability region only appears under limited supersaturation conditions; thus, growth hysteresis is unlikely to occur, suggesting that no OCZ is generated by this mechanism.

### Supplementary Information

Supplementary Information 1.

## Data Availability

The datasets used and/or analysed during the current study available from the corresponding author on reasonable request.
