# Supplementary material for: Oscillatory zoning of minerals as a fingerprint of impurity-mediated growth
Source: Sci Rep. 2024 Jun 20;14:13337. doi: 10.1038/s41598-024-63722-4 (PMC11190243; doi:10.1038/s41598-024-63722-4)
Supplement: Supplementary file 1 — Supplementary Information 1. [file 41598_2024_63722_MOESM1_ESM.pdf]

Supplementary Information

Oscillatory zoning of minerals as a fingerprint of impurity-mediated growth

Hiroki Torii[1] & Hitoshi Miura[1*]

[1]Graduate School of Science, Nagoya City University, Yamanohata 1, Mizuho-cho, Mizuho-ku, Nagoya, 46-78501, Aichi, Japan

[*] Corresponding author, miurah@nsc.nagoya-cu.ac.jp

Supplementary Figure 1 **Interdependence between the step velocity $\hat{V}_{\rm i}$ and adsorbed impurity density $\bar{\theta}$.** The solid line represents the relationship given by equation (3) and the dashed line represents the relationship given by equation (4). The difference in color represents the difference in supersaturation σ. We set $\hat{V}_0 = 5$ and $\hat{\kappa} = 1$.

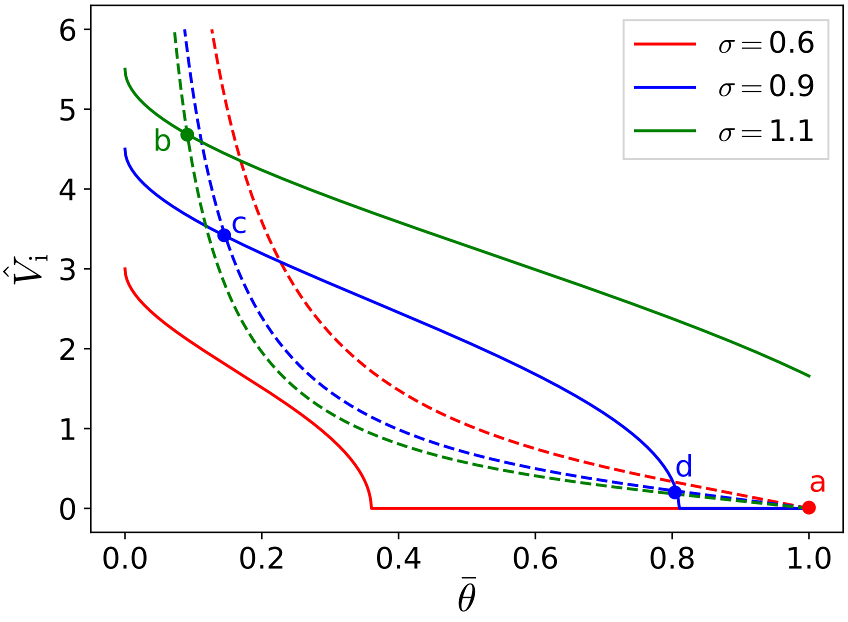

Supplementary Table 1 Values of parameters used in the calculations. When assuming impurities with slow adsorption (orange line in Figure 3), we used $\tau_{\mathrm{ad}} = 0.2$ sec and $\hat{V}_0 = 10$.

Parameter	Notation	Quantity
Diffusivity (solute)	D_{s}	1.0×10^{-9} (m^2/s)
Diffusivity (impurity)	D_{i}	0.3×10^{-9} (m^2/s)
Solute molar volume in crystal	v_{m}	2.6×10^{-5} (m^3/mol)
Solubility	c_{e}	1.0 (mol/L)
Initial concentration of solute	$c_{\mathrm{s}}(x, 0)$	2.2 (mol/L)
Initial concentration of impurity	$c_{\mathrm{i}}(x, 0)$	2.2×10^{-2} (mol/L)
Mean lifetime of ad-impurities	τ_{ad}	0.1 (sec)
Kinetic parameter	$\hat{V}_0$	5.0
Step edge free energy (normalized)	$\hat{\kappa}$	1.0
Step height	a	0.3 (nm)
Partition coefficient	K	1.0
Solution width	L	80 (mm)
Computational cell size	Δx	25 (μm)
Time increment	Δt	0.2 (sec)
